# The Effect of Continuous and Discretized Presentations of Concurrent Augmented Visual Biofeedback on Postural Control in Quiet Stance

**DOI:** 10.1371/journal.pone.0132711

**Published:** 2015-07-21

**Authors:** Carmen D’Anna, Maurizio Schmid, Daniele Bibbo, Maurizio Bertollo, Silvia Comani, Silvia Conforto

**Affiliations:** 1 Department of Engineering, Roma Tre University, Rome, Italy; 2 Department of Basic and Applied Medical Sciences, University “G. D’Annunzio”, Chieti, Italy; 3 Department of Neuroscience and Imaging, “G. d'Annunzio” University, Chieti, Italy; 4 Behavioral Imaging and Neural Dynamics Center BIND - “G. d'Annunzio” University, Chieti, Italy; University of California, Merced, UNITED STATES

## Abstract

The purpose of this study was to evaluate the effect of a continuous and a discretized Visual Biofeedback (VBF) on balance performance in upright stance. The coordinates of the Centre of Pressure (CoP), extracted from a force plate, were processed in real-time to implement the two VBFs, administered to two groups of 12 healthy participants. In the first group, a representation of the CoP was continuously shown, while in the second group, the discretized VBF was provided at an irregular frequency (that depended on the subject's performance) by displaying one out of a set of five different emoticons, each corresponding to a specific area covered by the current position of the CoP. In the first case, participants were asked to maintain a white spot within a given square area, whereas in the second case they were asked to keep the smiling emoticon on. Trials with no VBF were administered as control. The effect of the two VBFs on balance was studied through classical postural parameters and a subset of stabilogram diffusion coefficients. To quantify the amount of time spent in stable conditions, the percentage of time during which the CoP was inside the stability area was calculated. Both VBFs improved balance maintainance as compared to the absence of any VBF. As compared to the continuous VBF, in the discretized VBF a significant decrease of sway path, diffusion and Hurst coefficients was found. These results seem to indicate that a discretized VBF favours a more natural postural behaviour by promoting a natural intermittent postural control strategy.

## Introduction

Balance is one of the most studied abilities in motor control and learning. It is defined as the ability to maintain the centre of gravity of the body within its base of support. Posture is the stereotypical alignment of body/limb segments which allows body to maintain balance.

Maintaining balance through postural control is the main goal of postural tasks. Altough no generally accepted definition of postural control exists, the most widely used definition is the one given by Pollock and colleagues [1], who describe postural control as "the act of maintaining, achieving or restoring a state of balance during any posture or activity". Postural stability can be defined in terms of sway variability reduction, changes of the center of pressure (CoP) dynamics, other factors, or, finally, a combination of these. One of the most widely used definitions refers to the changes of the CoP dynamics, which are an indirect measure of postural sway (and thus a measure of a person’s ability to maintain balance) and can be derived from direct measures of the CoP.

Despite its seemingly simplicity, maintaining balance throughout postural adjustements in upright stance is a rather complex task that requires the integration of information coming from different sensory channels including proprioception, vestibulum, and vision [2]. Endogenous activities, such as respiration or hemodynamics, can be possible causes of disturbance [3, 4], and need to be counteracted to keep balance. As well, the functional deficit of one or multiple sensory channels [5], or the presence of neuromuscular diseases [6], may cause a diminished efficacy in balance control often resulting in modifications of postural oscillations. In hemiplegic patients, for instance, postural control is characterized by an increased postural sway [7, 8] and an asymmetrical body weight distribution that moves the CoP towards the unaffected side [9, 10].

Maintaining balance in quiet stance or in more dynamic conditions requires ongoing regulation provided through proprioceptive and exteroceptive intrinsic feedback [11]. Several studies have shown that the central nervous system stabilizes upright stance through two neural control strategies, which use either continuous [12] or intermittent [13, 14] feedback controllers to execute sustained or ballistic movements respectively. The control of sustained movements has been explained and interpreted within the framework of the continuous control theory and it has been often modeled by using a continuous disturbance signal [12, 15]. Loram and colleagues [13] have compared continuous and intermittent control by considering the effect of external stimuli and/or a perturbation on postural stability in quiet standing. They have further shown that event-driven intermittent control provides a framework to explain human behaviour under a wider range of conditions than continuous control, and that intermittent open loop action is a natural consequence of human physiology [13,14]. Interestingly, Gawthrop and colleagues [16] have also demonstrated that when event thresholds are small and sampling is regular, the continuous-time and intermittent controller cannot be distinguished. As a consequence, to differentiate the continuous and intermittent control systems, events driving control should occur at an irregular pace.

To evaluate the characteristics of postural stability, a variety of monitoring devices has been developed, such as video-based systems [17], accelerometers [18], and force plates [19]. In particular, force plate data have the advantage of being directly related to the reaction forces exerted by the ground, and thus they incorporate information on both the amount of unbalance and the mechanisms that are used to counteract it. Force plates are also employed in balance training and rehabilitation systems in populations differring for age and/or health conditions [20, 21], including the deterioration of cognitive competencies [22].

Balance can in fact be affected by cognitive processes such as the attentional focus [23], which can be driven by instructions and/or feedback provided to the performer. By leveraging this dependency, force plates are used in biofeedback systems [24, 25] to improve human motor control [26] by providing additional information related to the body motion or to its effect on some external elements, hence supplementing the natural sensory data [24, 25, 26]. This feedback is supplied in the form of audio [25], video [27], vibrotactile [28] or multimodal output [29]. It has been demonstrated that current feedback—as opposed to no feedback—facilitates the learning process. As well, in the constrained action hypothesis, it has been stated that an external focus of attention promotes a more automatic mode of control that uses unconscious, fast, and reflexive control processes [30, 31]. Therefore, instructions and feedback inducing an external focus of attention improve movement effectivenees and are beneficial for balance tasks [23]. Noteworthy, Shea and Wulf [32] argued that a visual feedback supplied on a computer screen provides a constant reminder to keep an external focus of attention, therefore facilitating balance maintainance. On the other hand, even in cases of feedback inducing an external focus of attention, conditions triggering neural activations in the self system would most probably result in a degraded performance [33].

Undoubtedly, vision is one of the most important sources of information to keep balance, as it permits to interact with the environment using exteroceptive feedback. Since it has been demonstrated that flows of visual information affect posture and balance [34], vision can be considered as a channel of concurrent augmented feedback from the environment.

In visual biofeedback systems (VBF), subjects typically stand on a force plate, and watch a computer screen where a representation of the position of their CoP is supplied in real-time. This type of concurrent augmented feedback can be used to control balance and regulate body sway in static or dynamic conditions [35]. Various modalities of VBF have been tested so far, the most straightforward one consisting in directly displaying the current position of the CoP [35–37]. Alternative methods include the use of predictive information [38] or the representation of the relative weight distribution on the platform [39].

The question whether VBF is beneficial to improve standing postural control is still controversial. A review on patient studies questioned the advantage of visual feedback therapy in bilateral standing compared with conventional therapy [6], whereas studies focussing on the effect of VBF on balance training and rehabilitation [24, 39] have demonstrated that postural performance improved [36] in people with disabilities or at-risk of falling, such as elderly people [27, 40]. Other studies performed in different populations (young, elderly, post-stroke patients) [27, 29, 40] have shown that the effect and the benefit of VBF on postural stability depends not only on the instructions given to the performer [41] but also on the adopted information representation: Cawsey et al. [37] demonstrated that the improvement of postural performance during quiet standing depended on the scale of the CoP visual display, whereas Rougier et al. [42] showed that variations in the time delay of the CoP presentation to the subject modulated postural behaviour.

Since the modalities of VBF presentation have different effects on the performer’s focus of attention [32], it may be of interest to investigate their possible impact on the control strategies adopted to maintain balance. Following this aim and in the light of the open questions described above, the purpose of our study was to: 1) compare postural stability during quiet upright stance in the absence and in the presence of VBF; 2) compare the effect on postural stability of a continuous and a discretized VBF presentation.

According to the *guidance* hypothesis [43, 44], in which feedback is claimed to guide the performer to the correct response, we expect that both types of VBF would improve postural control and balance maintainance as compared to the absence of VBF. Indeed, the absence of information given to the participants on the relationship between the feedback provided and their CoP, and the feedback provided on a computer display would induce an external focus of attention, which is considered the most effective one in terms of skill learning and performance, in agreement with Shea and Wulf [32].

With regard to our second study question, we hypothesize that the discretized VBF might be more efficient than the continuous one because it leads to a reduced feedback frequency. According to Wulf and Prinz [45], a reduction of the relative feedback frequency might give the subject a chance to perform the movement without being too concerned about movement execution, hence maintaining an external focus of attention directed on movement effectiveness. As a consequence, the detrimental effects of frequent feedback, which could divert the attentional focus on movement execution *per se* rather than on its effects, would be reduced. Furthermore, Wulf and Lewthwaite [33] have more recently suggested that conditions triggering neural activations in the self system would most probably result in a degraded performance. In our case, the degrading conditions might be determined by the type of feedback provided in the continuous VBF, i.e. the spot continuously moving inside a square, which, despite the absence of any specific information, can be easily associated with the changes of the CoP of the performer. Although this VBF would continue to induce an external focus of attention, it might implicitly act as a self-invoking trigger, inducing the performer to access self-schema that could deteriorate his/her performance.

The better influence of a discretized VBF on postural control is also expected on the basis of the results of prior studies on movement control performed within the framework of the theory of intermittent control [16], that includes cases when trigger frequency is irregular. Indeed, we anticipate that the intrinsic discontinuous nature of our discretized VBF modality might be more favorable than a continuous VBF for the maintenance of a natural intermittent postural control strategy.

## Materials and Methods

### Ethics statement

The experimental procedure was reviewed and approved by the Ethics Committee of the Applied Electronics Section of the Department of Engineering (Ref. # 03–014) before the study began. The participants in the study were instructed about the experimental procedure and gave written informed consent according to the Declaration of Helsinki.

### Participants

Twenty-four healthy young volunteers were recruited for the study (12 males: age 25 ± 3 yrs, height 1.72 ± 0.08 m, weight 75.1 ± 7.3 kg; 12 females: age 24 ± 2 yrs, height 1.65 ± 0.04 m, weight 63.1 ± 5.1 kg). None of them reported of any vestibular pathologies or of any neuropathies at the peripheral level. All participants had normal visual acuity and no colour blindness.

### Experimental set-up and procedure

During the experiments, the participants stood barefoot, feet together [46], on a force plate able to record CoP data (details in the following section), and were asked to maintain an upright natural posture with arms along the trunk. They were randomly assigned to either one of the two groups (balanced numerosity):

- *Group 1*: participants were asked to stand upright in the fixed task sequence (*noVBF*-*VBF*_*CoP*_)
- *Group 2*: participants were asked to stand upright in the fixed task sequence (*noVBF*-*VBF*_*emoticon*_).

This between-subject design was adopted instead of a randomized within-subject design to avoid the asymmetrical skill transfer effect, which would have resulted in a bias between conditions, even when randomizing the trials [47].

**Fig 1:**
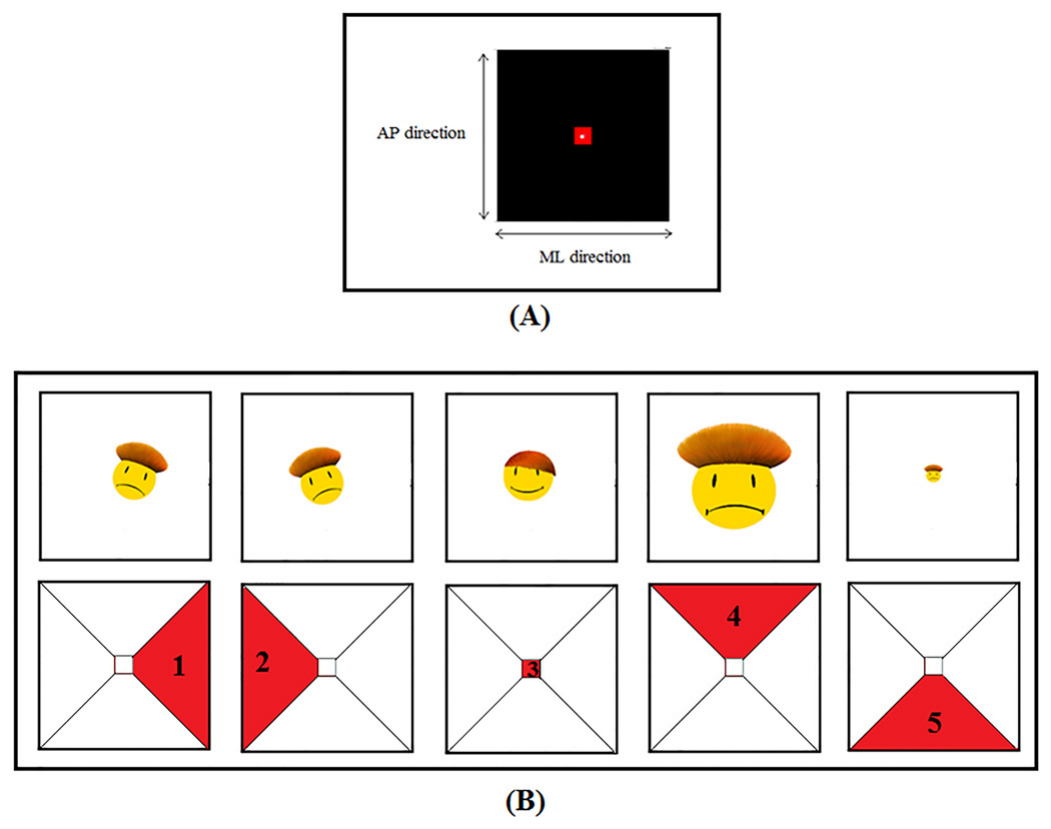
Modality of VBF presentation. (A) VBF_CoP_ presentation: the black square represents the force plate, the red square represents the area of stability, and the white spot represents the current position of the CoP. During tasks the spot moves in real time on the screen. (B) VBF_emoticon_ presentation: one out of five different emoticons appears in the centre of the screen. A smiling emoticon (3) appears if the participant stays in the stability area; if the CoP coordinates exceed the boundaries of the stability area, one out of a set of four sad emoticons is displayed. In particular: if the CoP exceeds the boundaries in the ML direction, the sad emoticon tilts 30° to the left (1) or to the right (2) hand side; if the CoP exceeds the boundaries in the AP direction, the sad emoticon is magnified (4) or reduced (5).

For both groups, each task type (*noVBF*, *VBF*_*CoP*_, *VBF*_*emoticon*_) was composed of three 40 s bouts. The inter-bout interval was 30 s. Each participant was allowed to familiarize with the VBF for a short period of time (less than 1 minute) before the experiment started. VBF was displayed on a 21'' LCD screen placed in front of the participants at a distance of 1 m. The instructions given to each participant before the experiment were:

- *noVBF (control condition)*: participants were asked to: “keep your visual focus (eye sight) on the display in front of you”. No instructions (which could induce a specific type of focus of attention) and no feedback were provided.
- *VBF*_*CoP*_: participants were asked to: “keep your visual focus (eye sight) on the white spot on the display, and try to maintain it inside the bigger red square”. This instruction induces an external focus of attention. The spot is a real-time representation of the CoP and the red square represents the stability area (Fig 1A), but no explicit information of this type is provided to the performer. Therefore, the instruction given for *VBF*_*CoP*_ and the continuous representation of the CoP support an associative external focus of attention [48]. The visual display reduced the amount of sway in the ratio 1:2 with respect to the physical area.
- *VBF*_*emoticon*_: participants were asked to: “keep your visual focus (eye sight) on the emoticon displayed on the screen, and try to maintain the smiling emoticon on”. Also this instruction induces an external focus of attention, and no explicit information is provided to the performer with regard to the reasons why emoticons change. In this VBF, the CoP data are used to control, in real-time, a set of five emoticons selected on the basis of the current CoP displacement: a smiling emoticon was displayed if the CoP position fell within the stability area (defined as in the VBF_CoP_ task); a sad emoticon was instead displayed if the CoP coordinates exceeded the stability area limits. Four different sad emoticons were used: two sad emoticons tilted by 30° to the left or to the right hand side were used if the subject’s CoP exceeded the boundaries in the medio-lateral (ML) direction, while two magnified or reduced sad emoticons were used if the subject’s CoP exceeded the limits in the antero-posterior (AP) direction (Fig 1B). Emoticons were chosen for this VBF for their popularity in everyday communication (e.g. computer mediated communication) [49].

In both VBFs the area of stability was computed a priori. It was fixed and set at 4x4 cm. The area of stability was fixed because differences in terms of anthropometric features (i.e height, length feet) were shown to not significantly affect CoP excursion measures [50].

### Data recording and processing

CoP displacements in the AP and ML directions were obtained from the signals recorded with a strain gage home-made force plate (40x40 cm, bandwidth 0–70 Hz, resolution 0.01 cm). Analogue signals were low-pass filtered (10 Hz) and sampled at 100 samples/s (NI USB-6210, by National Instruments). Upon sampling, a custom LabVIEW code (National Instruments Corporation) was used to control the VBFs in real time, and to store the CoP coordinates in the AP and ML directions for offline processing. Post-processing included mean value removal and digital low-pass filtering (cut-off frequency at 10 Hz [51]).

A subset of fifteen summary measures was extracted from the CoP time series, following the definition reported in [51, 52]:

- *Spatial Measures*: the standard deviation of the CoP in the ML and AP directions (STD_ML_, STD_AP_), the total length of sway path (SP), the mean amplitude (MA) representing the average distance of the CoP displacement from its mean value, the sway area (SA) estimating the area enclosed by the sway path per unit of time.
  As a metric to quantify the amount of time spent by the subject in stable conditions, the time percentage (T_%_) during which the CoP was inside the stability area was also calculated.
- *Frequency Measures*: the mean frequency (MF), defined as the rotational frequency of the CoP if it had travelled the total excursions around a circle with radius equal to the mean amplitude. It can be considered as a combined measure of sway excursion and frequency.
  From the density power spectrum of the AP and ML CoP time series, the mean power frequency in both directions was extracted (Mpf_ML_ and Mpf_AP_).
  Both spatial and frequency measures are used to quantify the relationship between postural control and stability.
- *Stabilogram diffusion coefficients*: from the stabilogram diffusion function obtained from the ML, AP and radial displacements of the CoP [52], the short-term diffusion coefficients D_MLs_, D_APs_ and D_rs_ and the exponential short-term coefficients (Hurst coefficients: H_MLs_, H_APs_ and H_rs_) were also calculated. These parameters are calculated based on the fractional Brownian motion model of the CoP displacement, which specifies the degree to which a trajectory is controlled. Typically the model highlights changes in the postural strategies: the diffusion coefficients D and the Hurst scaling exponent H indicate whether the trajectory is more or less controlled [52]. Differently from long-term, short-term coefficients have been shown to display a high value of Intraclass Correlation Coefficient [50, 53].

### Statistical analysis

Descriptive statistics was calculated for all parameters (STD_ML_, STD_AP_, SP, SA, MA, MF, Mpf_ML_, Mpf_AP_, D_MLs_, D_APs_, D_rs_, H_MLs_, H_APs_, H_rs_ and T_%_). For each participant group, all parameters were considered as dependent variables in a repeated measures Multivariate ANOVA, with Task Repetition and Task Type as main factors. If an effect on Task Type was obtained, a 2-way ANOVA test (paired samples) for the dependent variables was performed to calculate their contribution to the overall significance. To check for differences between the VBF tasks, a 2-way ANOVA (independent samples) was performed, again with Task Repetition and Task Type as main factors.

## Results

### noVBF-VBF_CoP_

In the first group (noVBF-VBF_CoP_), MANOVA showed a global significant effect for Task Type (p<0.001), whereas no significant effect of Task Repetition was present.

- *Spatial Measures*—ANOVA shows a significant effect of Task Type for all the CoP Spatial Measures: STD_ML_ (p<0.001), STD_AP_ (p<0.001), SP (p<0.001), SA (p<0.05), MA (p<0.001). Task Type also significantly influences the amount of time in which the participants were able to maintain the CoP within the stability area: T_%_ (p<0.05). We observed that, when VBF_CoP_ was presented, a decrease of standard deviation in both directions, sway area and mean amplitude occurred; sway path significantly increased. T_%_ increased by 9% as compared to the noVBF task. Numerical results are reported in Table 1.
- *Frequency Measures*—ANOVA shows a significant effect of Task Type for all the CoP Frequency Measures: MF (p<0.001), Mpf_ML_ (p<0.01), Mpf_AP_ (p<0.001). When VBF_CoP_ was presented, we observed an overall increase of the Frequency Measures. Numerical results are reported in Table 2.
- *Stabilogram diffusion coefficients*—ANOVA shows a significant effect of Task Type for all the Hurst exponents and the short term diffusion coefficient in the AP direction: D_APs_ (p<0.05), H_MLs_ (p<0.001), H_APs_ (p<0.01), H_rs_ (p<0.01); all coefficients increased when VBF_CoP_ was presented. No significant effect appeared for the other short term diffusion coefficients. Numerical results are reported in Table 3.

**Table 1:** noVBF-VBF_CoP_ comparison. Descriptive statistics (Group mean ± standard deviation) of spatial measures in noVBF- VBF_CoP_. ANOVA results are also reported (n.s: p>0.05, * p<0.05, **p<0.01, ***p<0.001).

	noVBF	VBF_CoP_	
STD_ML_ (m)	5.45E-03±1.35E-03	3.72E-03±9.98E-04	***
STD_AP_ (m)	1.33E-02±4.41E-03	9.60 E-03±1.88E-03	***
SP (m)	2.89E-02 ±0.59E-02	3.38E-02 ± 0.59E-02	***
SA (m^2^/s)	8.48E-05 ± 3.49E-05	6.63E-05 ± 2.39E-05	**
MA(m)	1.17E-02 ± 0.37E-02	0.8E-02 ± 0.1E-02	***
T_%_ (%)	86.33 ± 9.25	95.23 ± 3.34	*

**Table 2:** noVBF-VBF_CoP_ comparison. Descriptive statistics (Group mean ± standard deviation) of frequency measures in noVBF- VBF_CoP_. ANOVA results are also reported (n.s: p>0.05, *p<0.05, **p<0.01, ***p<0.001).

	noVBF	VBF_CoP_	
MF (Hz)	0.42 ± 0.09	0.64 ± 0.09	***
Mpf_ML_ (Hz)	0.23 ± 0.10	0.30 ± 0.12	**
Mpf_AP_ (Hz)	0.30 ± 0.09	0.51 ± 0.09	***

**Table 3:** noVBF-VBF_CoP_ comparison. Descriptive statistics (Group mean ± standard deviation) of stabilogram diffusion coefficients in noVBF- VBF_CoP_. ANOVA results are also reported (n.s: p>0.05, * p<0.05, **p<0.01, ***p<0.001).

	noVBF	VBF_CoP_	
D_MLs_ (m^2^/s)	1.38E-05 ± 9.27E-06	1.14E-05± 6.05E-06	n.s
H_MLs_	0.83 ± 0.06	0.87±0.049	***
D_APs_ (m^2^/s)	1.41E-04 ± 8.36E-05	1.83E-04 ± 8.27E-05	*
H_APs_	0.90± 0.04	0.97±0.02	**
D_rs_ (m^2^/s)	1.54E-04 ± 8.92E-05	1.91E-04 ± 9.22 E-05	n.s
H_rs_	0.89 ± 0.04	0.96 ± 0.02	***

### noVBF- VBF_emoticon_

In the second group (noVBF-VBF_emoticon_), MANOVA showed a global significant effect of Task Type (p<0.05), but no significant effect of Task Repetition.

- *Spatial Measures*—ANOVA showed a significant effect of Task Type for all the Spatial Measures, except the sway path: STD_ML_ (p<0.01), STD_AP_ (p<0.05), SA (p<0.05), MA (p<0.01) decreased when VBF_emoticon_ was presented. Task Type also caused a significant increase of T_%_ (p<0.05). Numerical results are reported in Table 4.
- *Frequency Measures*—ANOVA shows a significant effect of Task Type for all the CoP Frequency Measures: MF (p<0.001), Mpf_ML_ (p<0.05) and Mpf_AP_ (p<0.01) increased when VBF_emoticon_ was presented. Numerical results are reported in Table 5.
- *Stabilogram diffusion coefficients*—No significant effect of Task Type appeared for the diffusion and Hurst coefficients. Numerical results are reported in Table 6.

**Table 4:** noVBF-VBF_emoticon_ comparison. Descriptive statistics (Group mean ± standard deviation) of spatial measures in noVBF-VBF_emoticon_. ANOVA results are also reported (n.s: p>0.05, *p<0.05, **p<0.01, ***p<0.001).

	noVBF	VBF_emoticon_	
STD_ML_ (m)	4.94E-03±1.52E-03	4.06E-03±1.47E-03	*
STD_AP_ (m)	1.15E-02±2.54E-03	9.85E-03±2.32E-03	**
SP (m)	2.99E-02 ± 0.39E-02	3.09E-2 ± 0.30E-2	n.s
SA (m^2^/s)	7.21E05 ± 2.24E-05	5.97E-05 ± 2.24E-05	*
MA (m)	1.03E-2±2.31E-3	8.63E-03 ± 2.32E-03	**
T_%_ (%)	90.74±4.81	95.04±3.44	*

**Table 5:** noVBF-VBF_emoticon_ comparison. Descriptive statistics (Group mean ± standard deviation) of frequency measures in noVBF-VBF_emoticon_. ANOVA results are also reported (n.s: p>0.05,*p<0.05, **p<0.01, ***p<0.001).

	noVBF	VBF_emoticon_	
MF (Hz)	0.48 ± 0.11	0.59 ± 0.13	***
Mpf_ML_ (Hz)	0.21 ± 0.08	0.26 ± 0.09	*
Mpf_AP_ (Hz)	0.30 ± 0.11	0.39 ± 0.11	**

**Table 6:** noVBF-VBF_emoticon_ comparison. Descriptive statistics (Group mean ± standard deviation) of stabilogram diffusion coefficients in noVBF-VBF_emoticon_. ANOVA results are also reported (n.s: p>0.05, * p<0.05, **p<0.01, ***p<0.001).

	noVBF	VBF_emoticon_	
D_MLs_ (m^2^/s)	9.04E-06 ± 4.44E-06	8.12E-06± 6.47E-06	n.s
H_MLs_	0.79± 0.06	0.81±0.05	n.s
D_APs_ (m^2^/s)	1.05E-04 ± 4.86E-05	1.09E-04 ± 5.59E-05	n.s
H_APs_	0.89 ± 0.04	0.91±0.04	n.s
D_rs_ (m^2^/s)	1.14E-04 ± 5.07 E-05	1.18E-04 ± 5.71 E-06	n.s
H_rs_	0.88 ± 0.04	0.89 ± 0.04	n.s

### VBF_CoP_-VBF_emoticon_

With regards to possible differences between the two VBF tasks (VBFCoP and VBFemoticon), MANOVA showed a global significant effect of Task Type (p<0.01), and no effect of Task Repetition.

- *Spatial Measures*—Task Type causes a significant decrease of the sway path values (p<0.01) when the VBF_emoticon_ was presented, as compared to the VBF_CoP_ presentation.
- *Frequency measures*: The effect of Task Type appeared on Mpf_AP_ (p<0.01), where we observed a lower value in the VBF_emoticon_ condition than in the VBF_CoP_ one.
- *Stabilogram diffusion coefficients*—All the stabilogram diffusion coefficents were significantly affected by Task Type. D_MLs_, D_APs_, D_rs_, H_MLs_, H_APs_ and H_rs_ were lower in the VBF_emoticon_ than in the VBF_CoP_ task (p<0.001 for all).

**Fig 2:**
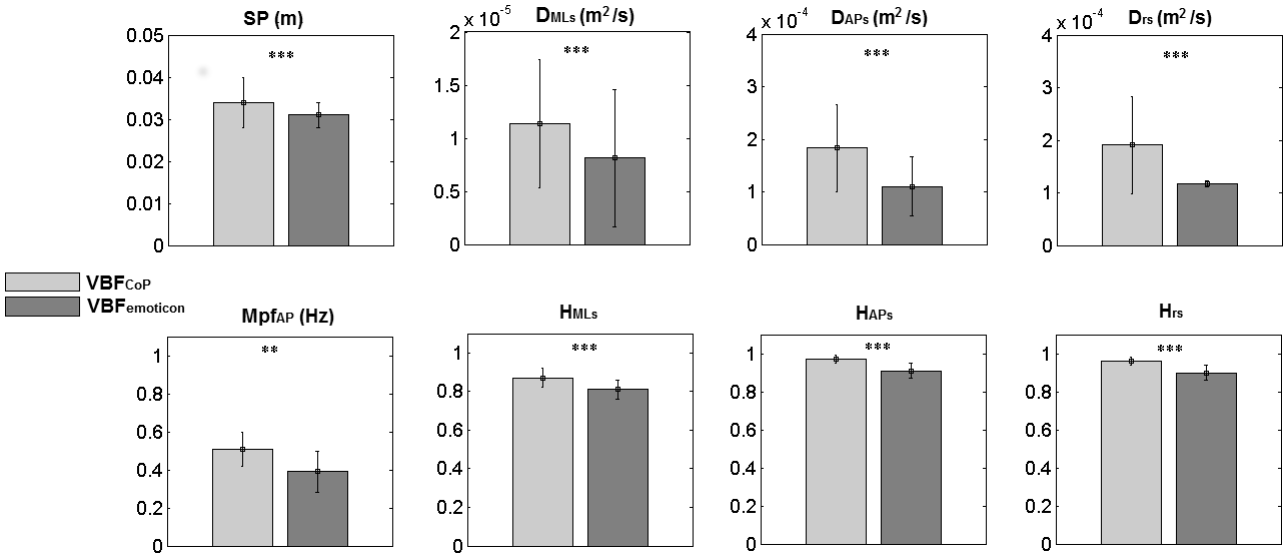
VBF_CoP_-VBF_emoticon_ comparison. Mean ± standard deviation for SP, Mpf_AP_, diffusion and Hurst coefficients in the two tasks (VBF_CoP_ and VBF_emoticon_). ANOVA result for Task Type are also reported (** p<0.01, ***p<0.001).

No significant difference was shown for the remaining parameters. Numerical results for the parameters showing a significant effect are given in Fig 2.

## Discussion and Conclusions

This study investigated the effect of VBF on postural stability during quiet upright stance with respect to the absence of any VBF, and the effect of two different VBF presentation modalities on postural performance. On one hand, the comparison of both VBFs (continuous and discretized) with the control condition (noVBF) showed, in both our study groups, that participants used VBF information to improve balance and postural performance during quiet standing, as assessed by a significant increase of the percent time spent in the area of stability and by a change of postural parameters. On the other hand, the comparison between the two VBFs showed that the modality of VBF presentation has an effect on postural behaviour: the significant difference observed in some postural parameters could indicate a change in the postural control strategies adopted, which might be influenced by the different frequency at which feedback was provided. All results will be discussed in detail below: we will first discuss the differences between each VBF and the control condition, and then we will focus on the differences between the two VBF presentations.

### The effect of continuous VBF on postural control

The presentation of the continuous VBF (VBF_CoP_) determined a general modification of all parameters. This change is in agreement with the results reported in relevant studies [27, 40]: young healthy adults are able to significantly reduce the excursion of sway, represented by sway area and mean amplitude, with a concurrent increase of the amount of time spent in the stability area. These changes are generally considered as indicators of an improved task performance [36], although a decrease in postural sway does not necessarily refer to an improvement in terms of postural performance at large [54]. This change in performance can be due to the presence of concurrent augmented feedback provided through a visual display, which induced an external focus of attention [32], generally recognized as beneficial for balance maintainance [23].

The presence of this VBF induced a corresponding increase of both sway path and mean frequency. A sway path increase generally indicates that the VBF determines a change in the postural control strategy, which has been associated with a moderate decline in the simple ankle strategy to maintain balance and with an increase of muscular activity [27, 42]. This interpretation is supported by the increase of the Hurst coefficients as compared to the control condition: the stochastic activity of the open-loop control mechanism diminishes and the CoP motion is more persistent, implying a reduced level of sensitivity to sensory information over short time scales [55].

### The effect of discretized VBF on postural control

The presentation of the discretized VBF (VBF_emoticon_) determines an effect on most, but not all, parameters. As for the continuous VBF presentation, we observed a reduction of both sway area and mean amplitude, and an increase of mean frequency. This outcome indicates that the concurrent augmented visual biofeedback has an effect on the subjects' dynamics during task execution, leading to an improvement of postural performance. Again, this might be due to the external focus of attention induced by the VBF and its presentation on a computer screen, which tend to keep the attention of the performer on movement effects rather than on movement execution *per se* [23, 32].

The absence of a significant effect on sway path might suggest that healthy young adults do not substantially modify their balance control strategy when the discretized VBF is supplied. This interpretation is confirmed by the absence of an effect on the stabilogram diffusion function coefficients. We thus speculate that the presentation of a discretized VBF, despite a modification of some parameters related to postural performance (i.e. SA, MA), does not induce a major change in the natural intermittent postural control strategy.

### Continuous VBF vs discretized VBF

The two VBFs present the same postural information in two different ways: in VBFCoP, postural information (CoP) is presented directly and continuously through the white spot moving on the screen, whereas in VBF_emoticon_ the same information is presented indirectly and in a discontinuous fashion through the expression of the displayed emoticon and its tilting/zooming.

When comparing the results obtained for the two VBF presentations (VBF_CoP_ and VBF_emoticon_), no significant differences were detected for the parameters reflecting sway excursion (those strictly related to task performance), whereas we observed significant differences for sway path, mean frequency of spectrum density power in AP direction, and diffusion and Hurst coefficients, which are associated with the type of postural control strategy. In particular, in VBF_emoticon_ the sway path is shorter, and the diffusion and Hurst coefficients are lower than in VBF_CoP_. These results might indicate that the VBF modality has a different impact on the postural control strategy adopted by the subjects in the two study groups. More specifically, the lower values of the stabilogram diffusion coefficients observed when a discretized VBF was presented might indicate that the open loop mechanisms—in both directions—contain a higher amount of stochastic activity as compared to the continuous VBF [42]. We might speculate that the continuous VBF facilitates the increase of muscular stiffness, hence a decrease of the stochastic activity level [52] and a less effective postural control.

The differences observed in the postural parameters between the two VBF modalities might also be related to the nature of the supplied VBF: in VBF_CoP_, feedback is provided continuously and is presented in a modality that can be easily related to the CoP position, whereas in VBF_emoticon_ feedback appears at a lower and irregular frequency and with a representation modality that cannot directly recall the CoP dynamics.

It has been claimed that conditions triggering neural activations in the self system would most probably result in a degraded performance [33]. These conditions may occur in the VBF_CoP_, where the feedback, provided through a direct representation of the CoP on the screen, can act, although not explicitely, as a self-invoking trigger. Indeed, this type of feedback can be easily associated with the performer's barycentre, which determines the changes of the CoP displayed on the computer screen. Therefore, these conditions would induce the performer to access self-schema that would affect his/her postural behaviour. Furthermore, considering that feedback, by its nature, implies an evaluation of an individual’s performance, it may not be surprising that frequent feedback can alter postural behaviour more than less frequent feedback [43]. Therefore, the high frequency with which feedback is provided in VBF_CoP_ would enhance the detrimental effects of the self-invoking trigger (represented by the type of feedback representation) by redirecting the focus of attention from movement effects (external focus) to movement execution *per se* (internal focus).

Another difference between the two VBF modalities regards the fact that in VBF_CoP_ the feedback frequency is constant, whereas in VBF_emoticon_ it is not. Therefore, on top of the beneficial effects of a lower feedback frequency, which reduces the risk for a recall to movement execution *per se*, it is possible that the irregular feedback frequency in the VBF_emoticon_ modality allows the performer to maintain an intermittent postural control strategy, which has been demonstrated to be a natural consequence of human physiology [14, 15, 16, 56]. Therefore, the results obtained with the use of the discretized feedback might suggest that this VBF presentation allows the motor system to self-organize more naturally, as a discretized VBF allows a “wait and act” strategy [57] with motor actions occurring only during short periods of time. According to Ikegami and colleagues [58], the presentation of intermittent visual feedback improves motor learning as it minimizes the deteriorating effect of the error feedback. By adopting this perspective, we can hypothesize that the continuous information provided in VBF_CoP_ could interfere with the natural intermittent control strategy, by providing an information flow (“energy in the loop” [14]) too high to be managed. As a consequence, the VBF_CoP_ could induce a less natural control mechanism than the discretized VBF, as the continuous presentation of feedback would reduce the recovery periods and subsequent execution of control actions, with consequent impairment of computational and muscular recovery [15]. Conversely, the intermittent provision of feedback typical of the VBF_emoticon_ would not interfere with the natural control strategy. Rather, VBF_emoticon_ would favor a more effective postural control by allowing the physiological recovery periods necessary for an efficient peripheral and central functioning.

We believe that our results and their interpretation could have important consequences for the knowledge of the mechanisms involved in postural control when dealing with biofeedbacks, as well as for the design and implementation of new balance training paradigms. According to this perspective, future studies based on a reinforced multidimensional and multimodal experimental protocol are needed. A complete analysis should include the monitoring of: a) the muscular activity (sEMG recording) to estimate modifications of stiffness and timing of muscular activation [59, 60], b) the capturing of the eye movements [61] to determine if and to what extent the subjects follow the information provided by the VBFs, and how this is mirrored in postural behaviour, c) psychometric evaluations of the focus of attention using, e.g., an adapted 11-point Borg scale [62]. Further advancements in the comprehension of the mechanisms of motor control should also include the monitoring of the EEG activity during balance tasks, to verify whether specific features of the continuous and intermittent control strategies are reproduced in the brain activations and/or in cortico-muscular coherence patterns [63], as well as the presentation of discretized VBF modalities without emotional valence, to verify whether the affective and/or emotional effect of the VFB modality has an influence on postural performance.
